# Why Kill the Cabin Boy?

**DOI:** 10.1017/S0963180120000420

**Published:** 2020-04-16

**Authors:** JOHN HARRIS

## Abstract

The task of combatting and defeating Covid-19 calls for drastic measures as well as cool heads. It also requires that we keep our nerve and our moral integrity. In the fight for survival, as individuals and as societies, we must not lose our grip on the values and the compassion that make individual and collective survival worth fighting for, or indeed worth having.^1^

The task of combatting and defeating Covid-19 calls for drastic measures as well as cool heads. It also requires that we keep our nerve and our moral integrity. In the fight for survival, as individuals and as societies, we must not lose our grip on the values and the compassion that make individual and collective survival worth fighting for, or indeed worth having.^1^

Recently in the United Kingdom, our doctors’ trade union The British Medical Association (BMA) and The National Institute for Health and Care Excellence (NICE), the latter established ostensibly to provide: “national guidance and advice to improve health and social care,” seem to be in danger of forgetting their values as well as ours, and their mission, their very *raisons d’être.*

On April 1, 2020, The BMA warned:

*“Health professionals may be obliged to withdraw treatment from some patients to enable treatment of other patients with a higher survival probability. This may involve withdrawing treatment from an individual who is stable or even improving but whose objective assessment includes a worse prognosis than another patient who requires the same resource.”* (https://www.bma.org.uk/media/2226/bma-covid-19-ethics-guidance.pdf.)

These suggested “obligations” were offered by the BMA to doctors via the BMA’s website. The idea was apparently to help doctors cope with the Covid-19 pandemic and the unpreparedness for it of the U.K. government. The statement is shockingly callous and also is almost certainly, as we shall see, both unethical and illegal. At the very least, it raises acute and complex questions of justice and law in a way that implies the answers to these questions are obvious, practical, and reflect a wide moral, social, and professional consensus. They are not, and do not.

A patient who is “stable or even improving” on treatment is clearly benefiting from that treatment, rendering treatment neither futile nor unnecessary. They may well be sick and even frail, but that is one obvious, and indeed compelling, reason why they are in need of care and treatment. If the treatment were to be withdrawn and the patient de-stabilized and their condition worsens, it would be difficult not to conclude that these consequences are highly likely to be attributable to the withdrawal of treatment, that is to say to result directly from the doctor’s actions.

When patients put themselves in the hands of their doctors, or, in the United Kingdom and elsewhere in Europe, in the hands of a public healthcare system, they rightly expect to be treated and are entitled to the best of their doctors’ and that system’s abilities. They do not expect to be abandoned by those doctors in favor of other patients with a higher survival probability, nor should they be so abandoned.

Another equally shocking example came from NICE. *The Independent* newspaper in the United Kingdom reported on March 25, 2020:

*“The* National Institute for Health and Care Excellence *(NICE) had been threatened with legal action for telling doctors they should assess patients with* learning disabilities, *autism* *and other limiting conditions as scoring high for frailty. But, following complaints from families and campaigners, NICE has said it will reissue its guidance. NICE’s national guidance for the NHS, published on Friday, advised doctors on how to choose who gets life-saving intensive care treatment such as ventilation in the event of critical care services being overwhelmed during a surge of Covid-19 patients. It included a frailty score that told hospitals to “assess all adults for frailty, irrespective of age and Covid-19 status” using a nine-point frailty scoring system with people “completely dependent for personal care, from whatever cause” scoring seven. Anyone scoring higher than five was said to have uncertainty around the benefits of critical care. Despite NICE accepting the guidance is wrong, at the time of publishing, it had yet to remove the incorrect documents from its website.*”

If a person is affected by a condition which grants them a life which they find worth living, be that condition what it may, then if illness or injury threatens, but there is a chance of restoring the *status quo ante*, or even improving on it, then they benefit to the same degree as anyone else who can be returned to their former life, or have that life enhanced through medical treatment or healthcare.^2^ Equally of course, they suffer if they are denied that chance of being restored to an existence they value.

A health care system or a professional body such as the BMA, which prioritizes “a higher survival probability,” or an influential advisory body such as NICE which formulates a points scoring system for eligibility for treatment, and which stipulates that “anyone scoring higher than five” would have doubtful benefits from critical care, are both, to put it politely, playing fast and loose with the concepts of “benefit” and of what constitutes health improvement. Even for a moment to include “*patients with* learning disabilities, autism, *and other limiting conditions as scoring high for frailty”* when NICE classes “frailty” as a treatment disqualifying factor, the effect is to remove ‘frail’ citizens from the ambit of moral and professional concern, to exclude them from the rule of rescue.^3^

To be sure, interventions confined to the easiest and cheapest to treat and to those most likely to recover, are well calculated to have impressive survival rates for their treatment regimes; successes perhaps second only to regimes that only accept patients who are entirely without injury or disease! No rational or halfway decent person will want or accept the terrible moral and human cost of abandoning those who need their assistance most; least of all those whom they have committed to treat by accepting them as patients or by assessing them in “Accident and Emergency,” the “Emergency Room” or clinic, and knowing that something can be done to improve their condition.

## The Doctrine of Necessity

The BMA and NICE seem to be appealing to what has been called “the doctrine of necessity”—that there would be literally “no choice” but to let someone worsen and probably die because other candidates for treatment might benefit more. This is deliberately to cause the death (or imperil the life) of one individual to save the life of another. But what, if anything, can make that other more deserving of treatment?

A landmark case concerning the doctrine of necessity established a precedent 136 years ago throughout the common law world, that necessity is no defense to a charge of murder. Moreover that it does not and cannot justify deliberately bringing about the death of one individual to preserve the life or lives of others. This case involved the deliberate and callous sacrifice of the cabin boy, the weakest of a party of starving shipwrecked sailors. In July 1884, Tom Dudley and Edwin Stephens were adrift in a tiny boat with two other survivors, the cabin boy Richard Parker and a fourth man Edmund Brooks. When the cabin boy fell into a coma and was much weakened and likely to die, Dudley and Stephens decided to kill him for food to save their own lives and that of Brooks (who took no part in the plan). They apparently believed they could not wait for him to die because killing Parker before his natural death would better preserve his blood to drink and hence give the others a better chance of survival. Not long after the cabin boy had been killed and eaten (probably 4 days later), the survivors were rescued. On return home, Dudley and Stephens were tried for murder and sentenced to hang; but were eventually reprieved (though not exonerated) and in fact served only 6 months in prison.

It is salutary to recall the words of the judgement of Lord Coleridge in this case:

*Lord Coleridge CJ:*

*"Now it is admitted that the deliberate killing of this unoffending and unresisting boy was clearly murder, unless the killing can be justified by some well-recognised excuse admitted by the law. It is further admitted that there was in this case no such excuse, unless the killing was justified by what has been called 'necessity'. But the temptation to the act which existed here was not what the law has ever called necessity. Nor is this to be regretted. Though law and morality are not the same, and many things may be immoral which are not necessarily illegal, yet the absolute divorce of law from morality would be of fatal consequence; and such divorce would follow if the temptation to murder in this case were to be held by law an absolute defence of it…"*

*"It is not needful to point out the awful danger of admitting the principle which has been contended for. Who is to be the judge of this sort of necessity? By what measure is the comparative value of lives to be measured? Is it to be strength, or intellect or what? It is plain that the principle leaves to him who is to profit by it to determine the necessity which will justify him in deliberately taking another's life to save his own. In this case the weakest, the youngest, the most unresisting, was chosen. Was it more necessary to kill him than one of the grown men? The answer must be 'No'.”* (*R v Dudley and Stephens* (1884) 14 QBD 273.)

When Lord Coleridge rhetorically and aptly asks: “Who is to be the judge of this sort of necessity? By what measure is the comparative value of lives to be measured?” he clearly echos Jeremy Bentham’s famous dictum that at the heart of both justice and democracy is the principle that “everybody is to count for one, nobody for more than one.” Bentham’s dictum, although implicit in his writings, comes to us from its guest appearance in John Stuart Mill’s essay “Utilitarianism.” This essay first appeared as a series of three articles published in *Fraser's Magazine* in 1861, which were collected and reprinted as a single book in 1863.^4^ It is therefore not entirely fanciful to suppose that Lord Coleridge was aware of Bentham’s dictum, and perhaps had it in mind when penning his judgement in Dudley and Stephens.

However that may be, it is this principle: that all persons matter equally, regardless of who or what they are; regardless of gender, race, color, nationality, religion, age, level of ability or disability. and all the rest; including vitally, health status and life expectancy. Abandon this principle and we are all, individuals and societies, faced with a myriad of invidious and arbitrary choices to which there are no agreed answers and no plausible ones neither.

As another English judge, Mars Jones J. said, in a more recent judgement and in a much cited case:

*However gravely ill a man may be…he is entitled in our law to every hour…that God has granted him. That hour or hours may be the most precious and most important hours of a man’s life. There may be business to transact, gifts to be given, forgiveness to be made, 101 bits of unfinished business which have to beconcluded. (R v Carr*, The Sunday Times, November 30, 1986.)

The implication of this judgement, and that in Dudley and Stephens, is that where choices have to be made between candidates for care, projected life expectancy or health state are neither legal nor moral bases for discrimination between people. Where all cannot be treated and priorities must be set, the basis of prioritization should not be based on personal characteristics, nor on their effect on the aggregate health of the whole community, for this will tend to discriminate against those, arguably most in need of health care.

If we were to attempt to translate this into a principle for the allocation of public resources to health care today, we might do worse than the following: “The principal objective of a public health care system should be to protect the life and health of each citizen impartially and to offer beneficial health care on the basis of individual need, so that each has an equal chance of flourishing to the extent that their personal health status permits.”^5^

Over 35 years ago in my book *The Value of Life*, I formulated a principle, which may have particular resonance here.

## The Value of Life Principle

*All of us who wish to go on living have something that each of us values equally, although for each it is different in character, for some a much richer prize than for others, and we none of us know its true extent. This thing is of course ‘the rest of our lives’. So long as we do not know the date of our deaths then for each of us the ‘rest of our lives’ is of indefinite duration. Whether we are 17 or 70, in perfect health or suffering from a terminal disease we each have the rest of our lives to lead. So long as we each wish to live out the rest of our lives, however long that turns out to be, then if we do not deserve to die, we each suffer the same injustice if our wishes are deliberately frustrated and we are cut off prematurely.* ^6^

As Mars Jones insisted, people value particular events within their life disproportionately to the time required to experience those events. Without having available the vast detail of each person’s life and their hopes and aspirations within that detail, we cannot hope to do justice between lives. The only sensible alternative is to count each life for one and none for more than one, whatever the differences in age and in other quality of life considerations. It is this outlook that explains why murder is always wrong, and wrong to the same degree, regardless of the age or health state of the victim. When you rob someone of life you take from them not only all they have, but all they will ever have. This is a difference in degree so radical that it makes for a difference in the quality of the act. However, the wrongfulness consists in taking from them something that they do not want to lose, and usually dread losing more than anything else they value.

## Selecting Between People for Rescue

Since for all of us, the value of the rest of our lives is for each person to determine for him or herself, then so long as we want to live that wish is sacrosanct. The value of life principle of course allows for selection between lives, but only in ways that give each life equal value. We humans have for millenia used such methods; they are familiar and well accepted.

These methods all involve ways of choosing without preferring (choosing between lives without doing so in a way that shows preference for the life or person chosen). *In extremis* drawing lots is one such method, the principle of first come first served is another, altruism, allowing people the option of giving away (if and only if they freely so choose) their equal priority to others is a third. Other methods, including the ones so recklessly advocated by the BMA, and espoused by NICE give opportunities for prejudice and arbitrary preference free rein.^7^

It is an extreme irony that the BMA, who have for so long resisted voluntary physician assisted death for *people who actually wish to die and who have good reasons to fulfil that wish*, should now be suggesting the legitimacy of withholding or withdrawing treatment from *people who have no wish to die.* In this crisis, we all need good fortune, but what we do not need or, I suggest want, is the dice to be deliberately loaded against us. We may be more ill than someone else, but if treatment is “stabilizing” or “improving” our condition, then treatment is by no means a waste of time or of resources, and all are entitled to an equal chance of receiving that treatment, even if all cannot actually receive it.

Bentham’s famous dictum sets out the basis for human rights and for equality before the law in any democratic jurisdiction. When Lord Coleridge delivered his judgement in 1884, he made a definitive statement, not only of the impossibility of arriving at a comparative value of different human lives, but also of the acute danger of attempting so to do, at least when those lives have reached an age at which they are protected by law. *"It is not needful to point out the awful danger of admitting the principle which has been contended for. Who is to be the judge of this sort of necessity? By what measure is the comparative value of lives to be measured? Is it to be strength, or intellect or what?”*

Unfortunately, it is needful in 2020 to point out to organizations such as the BMA and NICE, something that was obvious in 1884, namely the impossibility and undesirability of making judgements of comparative value between lives. Of course it is conceivable that in a democracy, such protections may be modified by a change in the Constitution (whether written, as in the United States, or not as in the United Kingdom) or possibly by Act of Parliament or the equivalent in other jurisdictions. What is sure, if anything is sure, is that it is, or should be, beyond the power of unelected acronyms like NICE so to do, and the same goes for unelected “initialisms” such as the BMA.

## Notes

1. I am indebted to my friend and colleague David Lawrence for very helpful comments on an earlier draft of this essay.

2.

Harris J. What is the good of health care? Bioethics 1996;10(4):269–92.1165655610.1111/j.1467-8519.1996.tb00129.x Harris J. QALYfying the value of life. Journal of Medical Ethics 1987;13(3):117123 [reprinted in Downie RS, ed. *Medical Ethics.* Aldershot: Dartmouth; 1996]10.1136/jme.13.3.117PMC13756583669036.

3. For a detailed discussion of the obligation of the State to deliver on the social contract and “the safety of the people,” see Harris J. How to be Good. Oxford and New York: Oxford University Press; 2016 [chap. 11].

4.

Mill JS . Utilitarianism In: Warnock M , ed. Utilitarianism. Fontana, London: William Collins Sons & Co. Ltd.; 1962.

5. See note 3, Harris 2016. See also note 2, Harris 1996.

6.

Harris J. The Value of Life. London: Routledge; 1985 [chap. 5], at 89.

7. It is my judgment that the discrimination involved in invoking the doctrine of necessity against the weak, the frail, and those with a poor (but not hopeless) treatment prognosis through age or reduced life expectancy, contravenes Articles 2 and 14 of the Human Rights Act 1998 and that the acts and omissions distinction is now so comprehensively demolished that it cannot be invoked in this (or any) context. But I have no space here to argue this further here. Human%20Rights%20Act%201998—Legislation.gov.uk.

