# Why Kill the Cabin Boy?—ERRATUM

**DOI:** 10.1017/S0963180120000705

**Published:** 2021-01

**Authors:** JOHN HARRIS

This guest editorial by Harris (2020)^1^ was originally published online as an article. It has now been reclassified as a guest editorial to the special section “Covid-19 Ethics: Principles and Policies.”

## Note

1

Harris, J. Why kill the cabin boy? Cambridge Quarterly of Healthcare Ethics 2020;30(1):4–9. 10.1017/S0963180120000420.32295664PMC7203162

